# Supplementary material for: Comparative Evolutionary Epidemiology of SARS-CoV-2 Delta and Omicron Variants in Kuwait
Source: Viruses. 2024 Nov 30;16(12):1872. doi: 10.3390/v16121872 (PMC11680180; doi:10.3390/v16121872)
Supplement: Supplementary file 1 [file viruses-16-01872-s001.zip › gisaid_supplemental_table_epi_set_240623oy.pdf]

SUPPLEMENTAL TABLE

Data Availability

GISAID Identifier: EPI_SET_240623oy
doi: 10.55876/gis8.240623oy

All genome sequences and associated metadata in this dataset are published in GISAID's EpiCoV database. To view the contributors of each individual sequence with details such as accession number, Virus name, Collection date, Originating Lab and Submitting Lab and the list of Authors, visit 10.55876/gis8.240623oy

Data Snapshot

- EPI_SET_240623oy is composed of 3,922 individual genome sequences.
- The collection dates range from 2019-12-26 to 2022-10-16;
- Data were collected in 189 countries and territories;
- All sequences in this dataset are compared relative to hCoV-19/Wuhan/WIV04/2019 (WIV04), the official reference sequence employed by GISAID (EPI_ISL_402124). Learn more at https://gisaid.org/WIV04.
