# Supplementary material for: Comparative Evolutionary Epidemiology of SARS-CoV-2 Delta and Omicron Variants in Kuwait
Source: Viruses. 2024 Nov 30;16(12):1872. doi: 10.3390/v16121872 (PMC11680180; doi:10.3390/v16121872)
Supplement: Supplementary file 1 [file viruses-16-01872-s001.zip › Table S1.pdf]

Table S1. Frequencies of the Delta and Omicron variants per selected discrete traits between between January 2021 and November 2022.

	**Collection year**		**Sex**			**Age**				**Collection**
	**2021**	**2022**	**Unknown**	**Female**	**Male**	**Unknown**	**50-20 years**	**< 50 years**	**< 20 years**	**by region**
Delta										
**Africa**	29	-	4	14	11	5	17	5	2	29
**Asia**	89	3	20	26	48	25	54	9	6	92
**Europe**	32	-	11	8	13	13	7	8	4	32
**Kuwait**	251	1	1	131	120	2	148	42	60	252
**North America**	41	-	16	12	13	15	13	9	4	41
**Oceania**	20	1	17	-	4	20	1	-	-	21
**South America**	26	1	14	11	2	10	9	6	2	27
**Totals**	**488**	**6**	**83**	**202**	**211**	**90**	**249**	**79**	**78**	**494**
Omicron										
**Africa**	5	30	2	14	19	2	19	9	5	35
**Asia**	-	49	13	17	21	16	19	12	4	49
**Europe**	-	26	7	11	8	8	8	8	2	26
**Kuwait**	-	383	-	170	213	21	244	74	44	383
**North America**	3	77	7	36	37	8	36	26	10	80
**Oceania**	-	22	4	6	12	22	-	-	-	22
**South America**	3	48	23	17	11	16	24	9	2	51
**Totals**	**11**	**635**	**56**	**271**	**321**	**93**	**350**	**138**	**67**	**646**
