# Supplementary material for: Comparative Evolutionary Epidemiology of SARS-CoV-2 Delta and Omicron Variants in Kuwait
Source: Viruses. 2024 Nov 30;16(12):1872. doi: 10.3390/v16121872 (PMC11680180; doi:10.3390/v16121872)
Supplement: Supplementary file 1 [file viruses-16-01872-s001.zip › Table S2.pdf]

Table S2. Bayes factor (BF) comparisons of the Delta and Omicron phylodynamic models using stepping-stone (SS) and path-sampling (PS) procedures. BFs based on the SS marginal likelihood (MLL) estimates are on the lower off-diagonal of the table, while BFs based on PS MLL estimates are on the upper off-diagonal. The best-fitting models are boldfaced for each variant.

	**Bayes Factor**							
**Model**	UCED+CP	UCED+EG	UCED+ExG	UCED+SG	UCLN+CP	UCLN+EG	UCLN+ExG	UCLN+SG
	Delta							
UCED[a]+CP[b]	—	42	-22	8	76	99	-8	63
UCED+EG[c]	50	—	-64	-34	34	57	-50	21
**UCED+ExG[d]**	**52**	**2**	**—**	**30**	**98**	**121**	**14**	**85**
UCED+SG[f]	5	-45	-47	—	68	91	-16	55
UCLN[g]+CP	32	-18	-20	27	—	23	-84	-13
UCLN+EG	51	1	-1	46	19	—	-107	-36
UCLN+EGx	-85	-135	-137	-90	-117	-136	—	71
UCLN+SG	25	-27	-27	20	-7	-26	110	—
	Omicron							
UCED[a]+CP[b]	—	-131	-168	-146	-70	-141	-147	-134
UCED+EG[c]	164	—	-37	-15	61	-10	-16	-3
**UCED+ExG[d]**	**169**	**5**	**—**	**22**	**98**	**27**	**21**	**34**
UCED+SG[f]	154	-10	-15	—	76	5	-1	12
UCLN[g]+CP	11	-153	-158	-143	—	-71	-77	-64
UCLN+EG	163	-1	-6	9	152	—	-6	7
UCLN+EGx	171	7	2	17	160	8	—	13
UCLN+SG	83	-86	-86	-71	72	-80	-88	—

[a]Uncorrelated relaxed clock with exponential distribution
[b]Constant population size coalescent model
[c]Expansion population size coalescent model
[d]Exponential population size coalescent model
[f]Bayesian skylgrid coalescent model
[g]Uncorrelated relaxed clock with log-normal distribution
